# First Report of Pembrolizumab Activity in KIT-Mutated Thymic Carcinoma

**DOI:** 10.3390/curroncol32020068

**Published:** 2025-01-27

**Authors:** Tommaso Martino De Pas, Giuseppe Giaccone, Chiara Catania, Fabio Conforti, Laura Pala, Periklis Mitsakis, Pierre-Yves Dietrich

**Affiliations:** 1 Division of Medical Oncology, Cliniche Humanitas Gavazzeni, 24125 Bergamo, Italy; chiara.catania@gavazzeni.it (C.C.); fabio.conforti@gavazzeni.it (F.C.); laura.pala@gavazzeni.it (L.P.); 2 Department of Hematology and Medical Oncology, Weill Cornell Medical College, New York, NY 10075, USA; gig4001@med.cornell.edu; 3 Nuclear Medicine, Hirslanden Clinique des Grangettes, 1224 Geneva, Switzerland; periklis.mitsakis@hirslanden.ch; 4 Medical Oncology, Hirslanden Clinique des Grangettes, 1208 Geneva, Switzerland

**Keywords:** thymic carcinoma, immunotherapy, KIT mutation

## Abstract

The antitumor activity of immunotherapy is strongly influenced by the presence of driver gene mutations/translocations. For this reason, knowledge of the predictive value of specific genetic alterations in relation to anti-PD(L)1 activity is highly useful for the clinical decision making process in many solid tumors, particularly in Non-Small Cell Lung Cancer. Although data on the correlation between genetic alterations and response to immunotherapy are available in the majority of common cancers, data are lacking in the subset of patients with KIT-mutated Thymic Carcinoma (TC). As a consequence, although immunotherapy is a standard treatment for TC patients, the lack of this knowledge leads to uncertainty when proposing immunocheckpoint inhibitors in this subset of patients. Here we describe the first report of a patient with KIT-mutated TC who received the anti-PD1 agent pembrolizumab, which caused a sustained partial response. This case report of a sustained partial response achieved with pembrolizumab in a patient with KIT-mutated TC after progression to chemotherapy and imatinib may be supportive during clinical decision making for this extremely rare disease.

## 1. Introduction

Thymic epithelial tumors (TETs) represent a very heterogeneous group of rare neoplasms with a complex histopathologic classification. Thymic Carcinoma (TC) is the most aggressive TETs subtype, showing a 5-year overall survival of approximately 30–55% [1,2]. Patients with advanced TC are usually treated with systemic therapies, comprising platinum-based chemotherapy and antiangiogenic agents, such as sunitinib and lenvatinib [3,4,5,6].

Moreover, immunotherapy has shown robust clinical activity when given either as a monotherapy [7,8,9,10] or in combination [11] and is considered one of the standard treatment options in many international guidelines.

The antitumor activity of immunotherapy is strongly influenced by the presence of driver gene mutations/translocations [12,13]. Many factors can be correlated with such a predictive value, making it difficult to predict the role of each mutation in influencing the response to immunotherapy.

As an example, very low levels of anti-PD(L)1 activity have been reported in patients with Non-Small Cell Lung Cancer (NSCLC) harboring the majority of driver alterations that are often associated with low mutational burden and a non-smoking history. However, high levels of antitumor activity have been found in patients with NSCLC with some specific genetic mutations, such as K-Ras and BRAF mutations [14].

Although data on the correlation between genetic alterations and response to immunotherapy are available in the majority of common cancers, data are lacking in Thymic Carcinoma (TC), leading to uncertainty when proposing immunotherapy in this subset of patients. Namely, mutations of KIT have been described in 10% of TC cases [15], and KIT inhibitors have been reported to be efficacious in these patients [16,17,18], showing proof of concept regarding its role in driving tumor growth and progression.

In the absence of data on the efficacy of immunocheckpoint inhibitors in patients with KIT-mutated TC, deciding whether or not to treat this subset of patients with these agents is difficult. Indeed, in the event of the foreseeable ineffectiveness of such therapy, we risk exposing patients to unwarranted toxicity, while a choice to abstain from such a proposal risks depriving patients of a therapy that has been shown to be effective in the overall population of TC patients.

Here we describe the first report of a patient with KIT-mutated TC who received the anti-PD1 agent pembrolizumab and experienced a sustained partial response, despite the presence of ASXL1 and DNMT3A mutations.

## 2. Case Report

A 65-year-old male patient with no relevant comorbidities but restless legs syndrome was diagnosed in May 2019 with stage Iva (8th TNM) Thymic Carcinoma. A whole-body CT scan and a fludeoxiglucose-18 positron emission tomography (PET) scan showed an anterior mediastinal lesion with vertebral invasion and pleural metastases. A biopsy was performed through a video-assisted thoracic surgery procedure, confirming the pleural tumor localizations.

A pleural biopsy showed TC-positive c-kit/CD117 staining, and an NGS-400 analysis found a KIT p.Trp557Arg mutation (38%, pathogenic). Other findings were the ASXL1 mutation p.Arg693Ter (47%), the DNMT3A mutations p.Ile634fs (41%) and p.Met329fs (47%), and TMB 3.8 non-silent mut/MB, PDL1 negative.

The patient was asymptomatic, with the exception of mild chest pain.

Chemotherapy with cisplatin, doxorubicin, and cyclophosphamide was given for seven cycles, with significant tumor shrinkage and resolution of thoracoalgia. Subsequently, palliative radiation therapy was administered to the vertebral T3 invasion (39 Gy, 13 fractions).

On June 2020, following pleural progression, the patient received imatinib 400 > 800 mg daily, on the basis of the presence of a mutation in the KIT gene potentially sensitive to KIT inhibitors. The treatment caused a partial response lasting 16 months, with a morpho-metabolic decrease and a decrease in size of the pleural and pulmonary lesions, without new lesions appearing.

In April 2022 a new biopsy was performed on a progressing pleural lesion. The NGS-400 analysis was superimposable to that of 2019, confirming KIT (38% allele frequency), ASXL1 (37% allele frequency), and DNMT3A (35% allele frequency) mutation; TMB was 10 non-silent mutations/1.458 Mbp = 6.86.

Pembrolizumab administration (200 mg q3wks) was started in August 2022. An FDG PET scan, performed immediately before treatment started, showed a left anterior mediastinal lesion with involvement of the diaphragm and pericardium, left para-scissural nodules, and a T5-paravertebral lesion. During pembrolizumab treatment, a planned radiotherapy session on T5 was conducted with palliative intent.

A new PET scan was performed after four cycles of immunotherapy, showing progression of all the previously identified lesions.

Due to the clinical symptoms’ improvement, namely pain resolution, pseudo-progression was assumed, and treatment with pembrolizumab continued.

**Figure 1:**
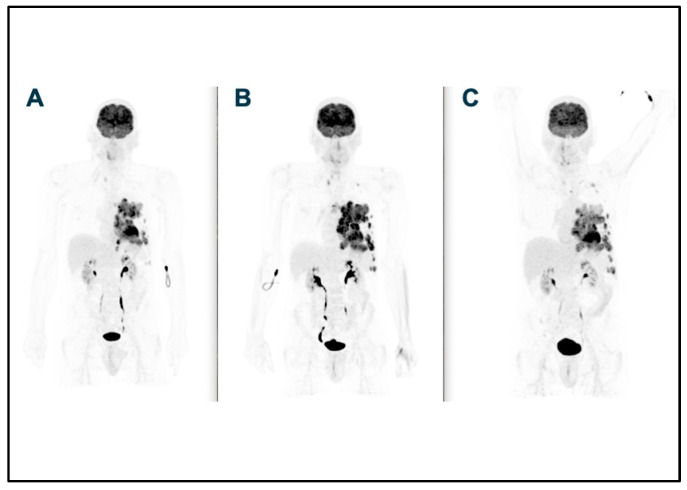
WB images of baseline ^18^F-FDG PET scan (**A**), ^18^F-FDG PET scan after first 4 cycles of immunotherapy (**B**), and subsequent ^18^F-FDG PET scan (**C**).

**Figure 2:**
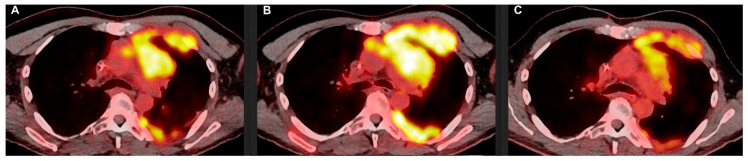
The left thoracic lesions with mediastinal, pericardial, pleural, and rib involvement in the baseline ^18^F-FDG PET/CT scan (**A**), as well as their evolution in the ^18^F-FDG PET/CT scan after the first 4 cycles of immunotherapy (**B**) and in the subsequent ^18^F-FDG PET/CT scan (**C**). SUXmax mediastinal mass and pleural lesion: A: 12.3 and 9.5; B: 13.2 and 13.7; C: 9.7 and 10.9.

A confirmatory PET scan performed after three additional cycles showed a tumor response from all the mediastinal and pleural metastases (Figure 1 and Figure 2), according to iRECIST [19], that lasted until August 2023, when the patient developed liver metastases.

Subsequently, lenvatinib was given, obtaining a tumor response lasting 5 months.

At present, a new chemotherapy regimen involving paclitaxel and carboplatin is planned.

## 3. Discussion

The use of immunotherapy in the treatment of patients with cancer has become part of the standard of care for many solid tumors, and many efforts have been made to identify the predictive factors of response and resistance to immunocheckpoint inhibitors. Among these, an important role is played by the presence of driver gene alterations, some of which have been shown to be negative predictors of response to immunotherapy, while others appear to have no influence on such antitumor activity. This knowledge is of paramount importance in daily clinical practice and is the basis of both cancer treatment guidelines and the selection criteria of many clinical trials concerning solid tumors, particularly in NSCLC.

The confirmed activity of pembrolizumab in patients with TC [7,8,9,10,11] has led to the inclusion of this agent in international guidelines, such as the NCCN guidelines, ESMO guidelines, and AIOM recommendations [20,21,22]. However, no data are available in the subset of patients with KIT-mutated TC.

KIT mutations seem to have a negative predictive value in many solid tumors in relation to anti-PD(L)1 activity. Patients with melanoma who developed KIT-mutated tumors received no survival benefit from anti-PD-1 adjuvant therapy compared with interferon treatment or observation in a real-world study [23]. Moreover, the therapeutic efficacy of immunotherapy in patients with KIT-mutated GIST falls short of expectations, despite the presence of abundant tumor-infiltrating immune cells in GIST patients [24].

The absence of data on PD(L)1 inhibitors’ antitumor activity in KIT-mutated TC makes it difficult for the oncology community to make a decision on whether or not to use these agents in this subset of patients.

This report of the sustained partial response achieved with pembrolizumab in a patient with KIT-mutated TC—despite the presence of ASXL1 and DNMT3A mutations—after progression to chemotherapy and imatinib may, therefore, be supportive during clinical decision making for this extremely rare disease.

## 4. Conclusions

Many treatments are routinely used for patients with advanced TC, such as chemotherapy, immunotherapy, and multi-TKI inhibitors.

Although the effectiveness of such therapies has been repeatedly confirmed in this patient population, their efficacy in patients with KIT-mutated TC has never been specifically reported. While there is no basis to speculate that the activity of chemotherapy could be compromised by this mutation, the possibility that immunotherapy may be less effective is theoretically justified by the evidence that some driver alterations reduce the efficacy of anti-PD(L)1 in other solid tumors, particularly in NSCLC.

This case is the first report of immunotherapy activity in a patient with advanced KIT-mutated TC progressing after systemic chemotherapy and imatinib. The sustained partial response achieved with pembrolizumab may be supportive of the choice of giving this agent to patients with this extremely rare disease. This report is in support of future prospective and retrospective analyses for the advancement of knowledge regarding this rare disease.

## Data Availability

Data available on request due to restrictions. The data presented in this study are available on request from the corresponding author.
